# Orbital Defect Size Reflects Structural Displacement but Not Ocular Motor Dysfunction: A Cohort Study of 1084 Patients

**DOI:** 10.3390/diagnostics16183043

**Published:** 2026-09-19

**Authors:** Tal Capucha, Ahmad Hija, Chaim Ohayon, Andrei Krasovsky, Amir Wolff, Omri Emodi

**Affiliations:** 1 Department of Oral and Maxillofacial Surgery, Rambam Health Care Campus, 8 HaAliya HaShniya Street, P.O. Box 9602, Haifa 3109601, Israel; capuchatal@gmail.com (T.C.); haim.ohayon@mail.huji.ac.il (C.O.); jim83carter@gmail.com (A.K.); amir.wolff@gmail.com (A.W.); omri.emodi@gmail.com (O.E.); 2 Ruth and Bruce Rappaport, Faculty of Medicine, Technion–Israel Institute of Technology, 1 Efron Street, Bat Galim, Haifa 3525433, Israel

**Keywords:** orbital fractures, computed tomography, defect area, enophthalmos, diplopia, ocular motility disorders, orbital floor, trauma severity

## Abstract

**Background/Objectives:** Orbital defect size is widely used as a surrogate for fracture severity, yet a larger bony opening and direct injury to the extraocular apparatus are not the same. We tested whether defect magnitude provides comparable information about structural displacement and ocular motor dysfunction. **Methods:** Retrospective cohort of CT-confirmed orbital fractures at a level 1 trauma center, 2001–2023. Repeated records were adjudicated to one index injury per patient, yielding 1084 patients. The estimated two-dimensional defect area (hereafter, defect area) was calculated from maximum sagittal and coronal dimensions and modeled per 50 mm^2^ using logistic regression, restricted cubic splines, covariate-standardized probabilities, and bootstrap area under the curve (AUC). **Results:** Median defect area was 143.9 mm^2^ (IQR 82.6–228.0). Per 50 mm^2^, adjusted odds increased for content herniation (odds ratio 1.37, 95% CI 1.28–1.48) and documented enophthalmos (1.45, 1.33–1.59) but not for extraocular movement abnormality (1.00, 0.92–1.09) or preoperative diplopia (1.01, 0.92–1.10). From 50 to 300 mm^2^, standardized probabilities rose by 35.9 and 26.3 percentage points for the structural phenotypes versus 0.2 and 0.6 for the functional phenotypes. Area-only AUCs were 0.670, 0.704, 0.500, and 0.508. **Conclusions:** Defect magnitude reflects structural displacement but shows little to no association with ocular motor dysfunction in this cohort.

## 1. Introduction

Orbital fracture assessment requires simultaneous interpretation of bony disruption, soft-tissue behavior, globe position, and ocular function. Conventional recommendations continue to cite substantial floor involvement, commonly more than half of the floor or an area near 2 cm^2^, along with clinical findings such as enophthalmos, diplopia, and tissue entrapment, as indications for reconstruction [1,2]. These criteria implicitly treat defect magnitude as a marker of overall fracture severity. However, the biological consequences of a larger bony opening are not equivalent to those of direct injury to muscle or connective tissue.

Recent evidence has exposed the limits of size-based reasoning. A systematic review of computed tomography (CT) parameters concluded that fracture size, location, orbital volume, soft-tissue involvement, and craniocaudal dimensions each provide distinct information and are more useful in combination than in isolation [3]. A critical review of two decades of literature found that reported size cut-offs varied substantially and were only weakly predictive of symptomatic fractures, whereas inferior rectus displacement was more consistently associated with symptoms [4]. Conversely, a meta-analysis of 36 studies confirmed that fracture area and changes in orbital volume are useful predictors of post-traumatic enophthalmos, linking approximately 3.12 cm^2^ of fracture area to 2 mm of enophthalmos [5]. These apparently divergent conclusions are reconcilable if defect size performs differently depending on the phenotype being predicted.

Small series have reported elements of this structural-functional dissociation. Ploder et al. found that fracture area was associated with enophthalmos but not with limitation of ocular motility in 38 isolated floor fractures [6]. Kunz et al. reported a correlation between defect size and globe position but not with diplopia or motility among conservatively managed patients [7]. Others have shown that soft-tissue herniation, herniation height, extraocular muscle position, and orbital volume change can be more informative than area alone for functional symptoms [8,9,10,11,12,13]. This literature is heterogeneous, usually restricted to isolated fractures, and generally underpowered to compare several structural and functional phenotypes within one cohort.

The purpose of this study was to determine whether the magnitude of orbital defects provides comparable information about structural displacement and ocular motor dysfunction in a large, unselected cohort of orbital fractures. We hypothesized that increasing estimated defect area would be strongly associated with orbital content herniation and globe-position abnormality, and substantially less so with extraocular movement (EOM) abnormality and preoperative diplopia.

## 2. Materials and Methods

### 2.1. Study Design and Setting

This retrospective observational cohort study used the institutional orbital fracture registry of the Department of Oral and Maxillofacial Surgery at Rambam Health Care Campus, a level 1 tertiary trauma center in Haifa, Israel. The registry includes CT-confirmed orbital fractures managed from September 2001 to November 2023. Reporting was guided by the STROBE statement [14] (Tables S1–S3).

### 2.2. Data Structure and Unit of Analysis

The registry is organized by case record, and a single orbital injury may generate more than one row. The source file contained 1489 rows, which were reduced to 1463 after removing 26 exact duplicates, representing 1084 unique patients. Repeated rows were therefore adjudicated before analysis rather than treated as independent observations: rows sharing a patient identifier were assigned to the same injury if they had the same recorded age or dates within 90 days of one another, and rows recording opposite orbits on the same date were treated as one bilateral injury. This yielded 1103 distinct orbital injuries. The analytic unit was the index (earliest) injury for each patient, yielding 1084 observations, of which 1083 had measurable defect dimensions. An analysis of all 1103 injuries with patient-level clustering was retained as a sensitivity analysis.

### 2.3. Defect Morphometry and Covariates

Maximum sagittal and coronal defect dimensions were recorded in millimeters from multiplanar facial CT. All defect dimensions were measured directly by the study team from the original CT images acquired at our institution, using the Sectra IDS7 Workstation picture archiving and communication system (PACS), version 28.1.4.10543 (Sectra AB, Linköping, Sweden), rather than extracted from prior radiology reports. The estimated two-dimensional (2D) defect area—hereafter, defect area—was calculated as an ellipse, (π/4) × sagittal × coronal, and modeled continuously for each 50 mm^2^ increase; this planar approximation is well established for isolated orbital floor defects, and its limitations for complex multi-wall fractures are addressed in the Discussion. Records with 0 × 0 mm entries were treated as non-measurable sentinel values rather than true zero-area defects. When records for a single injury conflicted, the largest recorded dimension was used rather than an average, on the basis that a partial or obliquely angled measurement is more likely to underestimate than to overestimate the true defect. The fracture pattern was grouped as isolated orbital floor, floor with inferior orbital rim, floor with medial wall, complex multi-wall, or other. Adjustment covariates were age, sex, fracture pattern, mechanism of injury (fall, assault, road traffic, sport, ballistic, workplace, and other), and the presence of an associated facial fracture.

### 2.4. Structural and Functional Phenotypes

Four prespecified primary phenotypes were analyzed. The structural phenotypes were CT-documented orbital content herniation and documented enophthalmos; the functional phenotypes were documented EOM abnormality and preoperative diplopia. Per departmental and institutional protocol, every patient with a CT-confirmed orbital fracture is evaluated by both the maxillofacial surgery team and ophthalmology (oculoplastics) before any intervention, with a further joint evaluation after intervention for operated patients; diplopia is registered only when both specialties concur. EOM abnormality and preoperative diplopia, therefore, reflect this dual-specialty, concordance-based assessment rather than a single examiner’s impression, although the registry does not capture a standardized gaze-specific grading scale, forced-duction testing, or the exact examination interval in hours or days. Enophthalmos is assessed within this same dual-specialty evaluation structure, but because the registry does not standardize the timing of enophthalmos assessment beyond it, that outcome is termed documented enophthalmos rather than late or postoperative enophthalmos: entries were abstracted from clinical and imaging documentation created at any point from the initial (preoperative) joint evaluation through postoperative follow-up, and because acute periorbital edema and retrobulbar hematoma can obscure true globe position in the immediate post-injury period, documented enophthalmos in this cohort should be read as a composite of early and late findings rather than as a measure of stable, late post-traumatic enophthalmos. Hypoglobus was analyzed as a secondary globe-position phenotype because it was not recorded across the entire cohort: it was marked not applicable in 402 records, all from non-operated patients, and those observations were treated as missing rather than negative.

Three coding conventions in the registry required explicit handling and were reported rather than resolved silently. Two records documented exophthalmos, the opposite of the phenotype of interest, and were not counted as enophthalmos. Ten records documented hyperglobus, which was likewise not counted as hypoglobus. Seven records had an uninterpretable mark in the enophthalmos field and were treated as missing rather than assigned to either category. Postoperative diplopia trajectories were deliberately excluded from this analysis and are reported separately.

### 2.5. Statistical Analysis

Continuous variables were summarized as medians and interquartile ranges (IQR), and categorical variables as counts and percentages. Because the analytic cohort contains exactly one observation per patient, associations were estimated using logistic regression; population-averaged generalized estimating equations with patient-level clustering and robust covariance were used only in the all-injury sensitivity analysis. Each model included estimated defect area, age, sex, fracture pattern, associated facial fracture, and mechanism of injury. Adjusted odds ratios (OR) with 95% confidence intervals (CI) and two-sided *p*-values are reported, with Benjamini–Hochberg false discovery rate correction applied across the four primary phenotypes.

To avoid imposing a linear exposure-response assumption, the defect area was also modeled with restricted cubic splines, and overall and nonlinearity likelihood ratio tests were computed against models without area and with linear area, respectively. Adjusted probabilities were standardized over the observed covariate distribution at fixed defect areas, with 95% CIs obtained by the delta method. We summarized discrimination attributable to defect area alone by the area under the receiver operating characteristic curve (AUC), with percentile intervals from 2000 bootstrap replicates. These AUCs quantify the information in a single continuous variable and do not evaluate diagnostic accuracy against a reference standard; no diagnostic or prediction model was developed, and STARD and TRIPOD are therefore not applicable. Prespecified sensitivity analyses examined a conventional 200 mm^2^ threshold, isolated floor fractures, patients contributing a single registry record, and all adjudicated injuries with clustering. A further sensitivity analysis, added post hoc at revision, stratified the cohort by scan era (2001–2010 versus 2011–2023), tested an area-by-era interaction by likelihood ratio, and refitted the primary models with scan era as an additional covariate. Missing data were not imputed. All statistical analyses were performed using IBM SPSS Statistics, version 32.0 (IBM Corp., Armonk, NY, USA).

## 3. Results

### 3.1. Cohort and Defect-Size Distribution

The analytic cohort comprised 1084 patients, each contributing one index orbital injury; 1083 had measurable defect dimensions. The median age was 31 years (IQR 22–50). A total of 865 patients (79.8%) were male. A total of 655 injuries (60.4%) involved the orbital floor in isolation, and 219 (20.2%) had an associated facial fracture. The median estimated defect area was 143.9 mm^2^ (IQR 82.6–228.0). Characteristics across defect-area quartiles are shown in Table 1. Structural findings increased steeply with size: content herniation from 34.8% in the smallest quartile to 72.0% in the largest and documented enophthalmos from 4.0% to 30.6%. Functional findings did not increase: EOM abnormality was 17.6% and 17.0%, and preoperative diplopia 16.5% and 18.1% in the smallest and largest quartiles, respectively.

### 3.2. Primary Adjusted Associations

**Figure 1:**
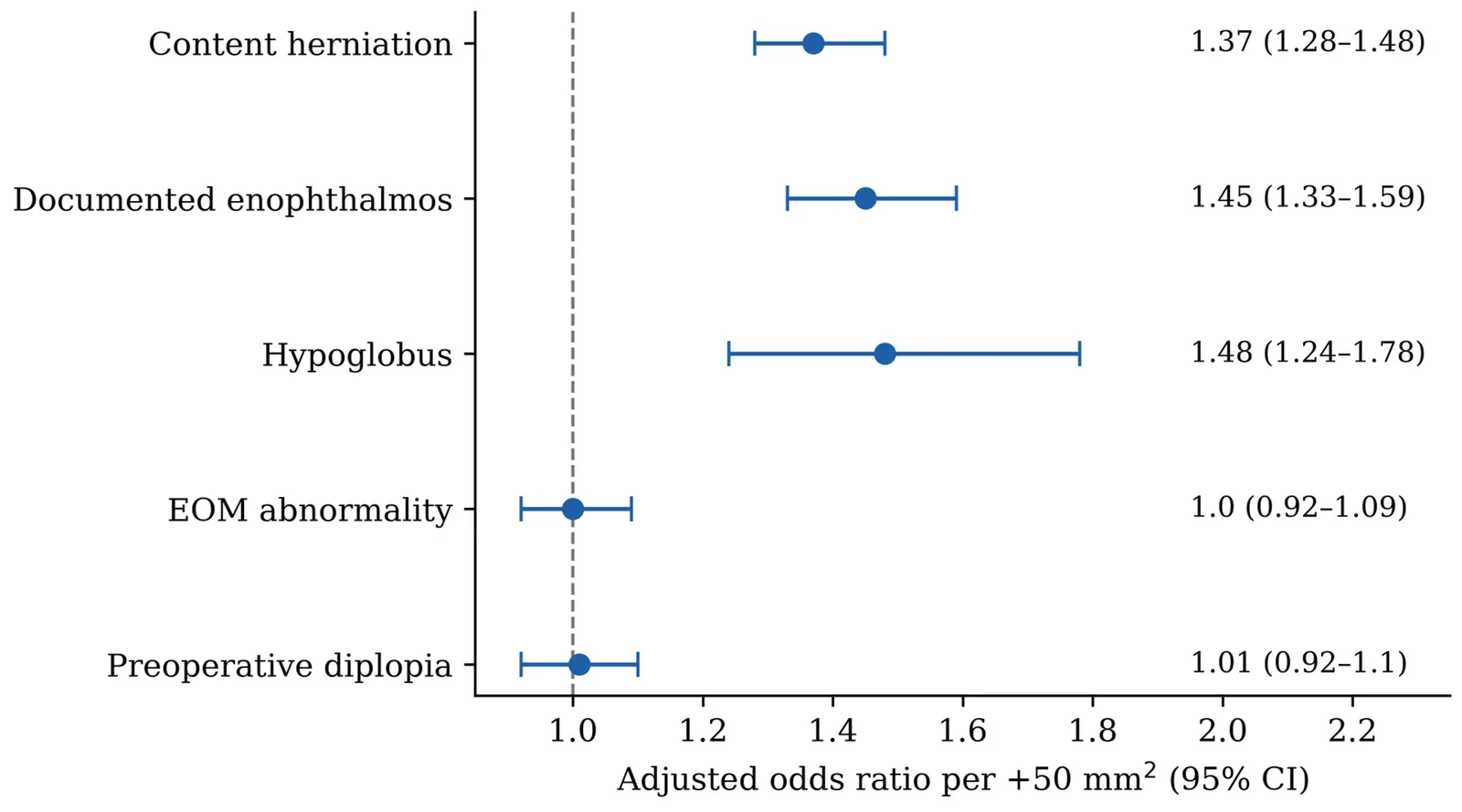
Adjusted odds ratios per 50 mm^2^ increase in estimated orbital defect area, with 95% confidence intervals. The dashed line marks the null.

Each 50 mm^2^ increase in estimated area was associated with 37% higher adjusted odds of content herniation (OR 1.37, 95% CI 1.28–1.48; *p* < 0.001) and 45% higher adjusted odds of documented enophthalmos (OR 1.45, 1.33–1.59; *p* < 0.001). Hypoglobus, analyzed secondarily, was also associated with increasing area (OR 1.48, 1.24–1.78; *p* < 0.001). In contrast, area was not associated with EOM abnormality (OR 1.00, 0.92–1.09; *p* = 0.944) or with preoperative diplopia (OR 1.01, 0.92–1.10; *p* = 0.849). The structural associations remained significant after false discovery rate correction, whereas the functional associations remained null (Table 2, Figure 1).

### 3.3. Standardized Probabilities and Non-Linearity

Restricted cubic splines showed strong overall associations between defect area and content herniation (overall *p* < 0.001; non-linearity *p* < 0.001) and between defect area and documented enophthalmos (overall *p* < 0.001; non-linearity *p* = 0.032). No overall association was observed for EOM abnormality (*p* = 0.355) or preoperative diplopia (*p* = 0.060); the non-linearity term for diplopia reached nominal significance (*p* = 0.025) in the absence of an overall association. In the context of five phenotypes tested, this is most plausibly noise.

**Figure 2:**
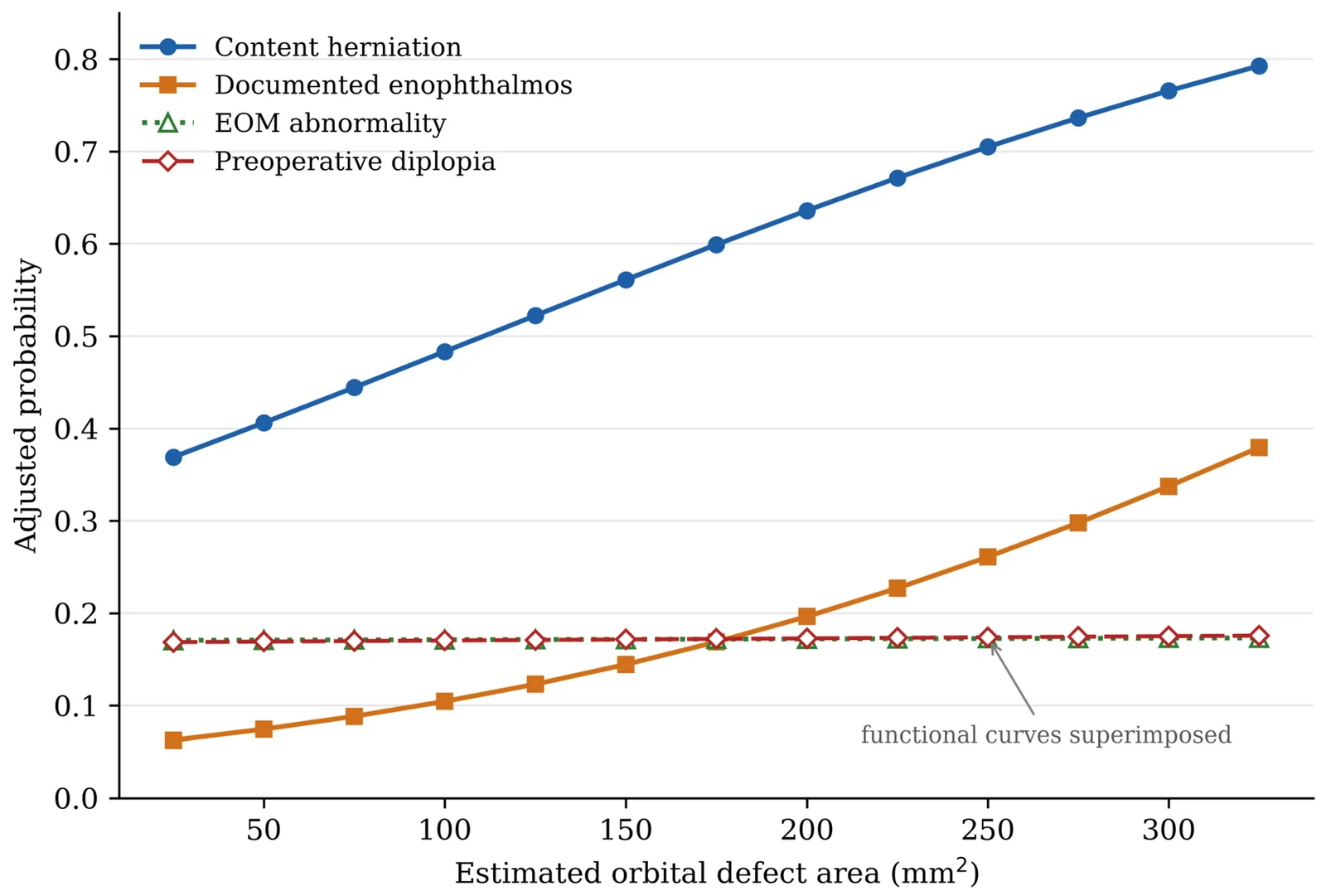
Covariate-standardized adjusted probability of each phenotype across estimated orbital defect area. Structural phenotypes rise steeply with defect area; the two functional phenotypes are flat and superimposed on one another.

Standardized probabilities indicate the magnitude of the divergence. Increasing the area from 50 to 300 mm^2^ raised the adjusted probability of content herniation from 40.6% to 76.6%, a risk difference of 35.9 percentage points (95% CI, 28.5–43.4), and the adjusted probability of documented enophthalmos from 7.4% to 33.7%, a difference of 26.3 percentage points (20.0–32.6). Over the same range, EOM abnormality increased from 17.1% to 17.3% (0.2 points; −5.8 to 6.2), and diplopia increased from 16.9% to 17.5% (0.6 points; −5.5 to 6.6) (Figure 2).

### 3.4. Discrimination Attributable to Defect Area

The defect area alone discriminated moderately for structural phenotypes and no better than chance for functional ones. The AUC was 0.670 (95% CI 0.636–0.703) for content herniation, 0.704 (0.662–0.740) for documented enophthalmos, and 0.719 (0.635–0.799) for hypoglobus, compared with 0.500 (0.455–0.544) for EOM abnormality and 0.508 (0.460–0.553) for preoperative diplopia.

### 3.5. Sensitivity Analyses

The separation remained stable throughout. At the conventional 200 mm^2^ threshold, adjusted odds were increased for content herniation (OR 2.51, 95% CI 1.90–3.31), documented enophthalmos (2.53, 1.83–3.51), and hypoglobus (4.12, 1.99–8.54), but not for EOM abnormality (1.10, 0.78–1.56) or diplopia (1.16, 0.83–1.63). Among isolated floor fractures, the pattern was reproduced (herniation 1.42, enophthalmos 1.40, EOM 1.01, and diplopia 1.04 per 50 mm^2^), as it was among patients contributing a single registry record and in the analysis of all 1103 adjudicated injuries with patient clustering (Table 3). Stratification by scan era reproduced the same separation in both subperiods. Among injuries imaged in 2001–2010 (n = 392), adjusted odds per 50 mm^2^ were 1.32 (95% CI 1.16–1.49) for content herniation and 1.47 (1.27–1.71) for documented enophthalmos, against 0.94 (0.80–1.12) for EOM abnormality and 1.05 (0.89–1.22) for diplopia; among injuries imaged in 2011–2023 (n = 691), the corresponding estimates were 1.44 (1.31–1.59), 1.47 (1.31–1.65), 1.05 (0.95–1.17) and 1.01 (0.91–1.13). No area-by-era interaction approached significance for any phenotype (all *p* ≥ 0.35), and adding scan era as a covariate to the primary models changed no estimate materially (Table 3).

## 4. Discussion

Orbital defect magnitude does not serve as a global measure of fracture severity. Across 1084 patients, increasing defect area was strongly and reproducibly associated with orbital content herniation and globe-position abnormality, whereas associations with EOM abnormality and preoperative diplopia were centered almost exactly on the null. The same separation was evident in absolute standardized risks, spline models, AUC analyses, and across all sensitivity cohorts. Defect size carried substantial information about structural displacement and showed little to no association with ocular motor dysfunction in this retrospective cohort.

The structural component aligns with the established relationship among defect magnitude, orbital volume change, and enophthalmos. Lentskevich et al. synthesized 36 studies and confirmed that fracture area and orbital volume change predict post-traumatic enophthalmos, estimating that approximately 3.12 cm^2^ of fracture area corresponds to 2 mm of enophthalmos [5]. Earlier CT studies linked greater defect area or displaced tissue volume to increased globe recession [6,13]. These data extend that evidence across a full-spectrum cohort and show that the adjusted probability of enophthalmos rises from 7.4% at 50 mm^2^ to 33.7% at 300 mm^2^. Because the registry does not standardize the timing of assessment, this should not be interpreted as a model of late enophthalmos.

The functional findings help reconcile inconsistent prior literature. Kunz et al. found a good correlation between defect size and globe position but not with diplopia or motility in 48 conservatively managed fractures [7]. Ploder et al. likewise found no correlation between fracture area and motility limitation in 38 isolated floor fractures [6]. Shah et al. showed that small and medium floor fractures with soft-tissue herniation could produce more diplopia than larger fractures without it [8], and Bruneau et al. found the floor-area ratio predictive of enophthalmos, whereas herniation height was more informative for diplopia [9]. Taken together with the present data, bony dimensions and functional ocular injury are related but non-equivalent domains.

A plausible mechanical account is that the size of the bony opening determines the potential for tissue displacement and volume change, whereas ocular motor dysfunction depends on the behavior and integrity of the extraocular muscles and their connective-tissue apparatus. Basta et al. reported that orbital volume increase and inferior rectus displacement were more informative than area alone for symptom development [10], and Furuta et al. related motility restriction to muscle position rather than fracture size [11]. Oku et al. similarly identified muscle-related rather than size-related factors as associated with diplopia before and after repair [12]. A defect can be large without deforming the muscle and small while incarcerating it.

This dual sensitivity offers a mechanistic explanation for the flat discrimination observed here, rather than a simple absence of effect. If small, so-called trapdoor defects disproportionately entrap the inferior rectus muscle or its fascial sleeve with little bony displacement, while large defects disproportionately produce diffuse contusion, hemorrhage, and edema of the extraocular muscles or their innervation regardless of the size of the bony opening, then entrapment risk and contusion/edema risk move in opposite directions across the range of defect area. Averaged across a cohort, these two opposite, size-discordant mechanisms would cancel any monotonic size-outcome gradient—precisely the pattern observed: flat restricted cubic splines, near-superimposed probability curves for EOM abnormality and diplopia, and AUCs indistinguishable from 0.50. A flat area-outcome curve, therefore, does not indicate that the extraocular apparatus is unaffected by defect size; it indicates that two opposing, size-dependent injury mechanisms are being averaged into a single null association. Two recent series point the same way. In 155 patients, preoperative diplopia was associated on multivariable analysis with the anatomical region of the fracture, in particular the middle and posterior thirds of the orbital floor [15]; and in a 14-year series of pure blowout fractures, fracture type rather than fracture size emerged as the dominant independent predictor of diplopia [16].

The clinical implication is narrow but practical. A large defect should raise concern about volume-related sequelae, that is, herniation, enophthalmos, and hypoglobus, and the AUCs of about 0.70 indicate that the area carries real but modest information even for those. A large defect should not by itself raise concern about motility, and, more importantly, a small defect should not be taken to exclude it: at 50 mm^2^ the adjusted probability of EOM abnormality was 17.1%, essentially identical to the 17.3% at 300 mm^2^. Motility must always be assessed clinically, regardless of defect size. This is not evidence that CT is uninformative about the extraocular apparatus: cross-sectional imaging remains essential for identifying muscle position, soft-tissue herniation, and fracture configuration. What the present data show is narrower and, we believe, more clinically useful: estimated defect area alone is not a meaningful predictor of ocular motility disturbance, and this null association should not be extended into a claim that CT findings in general are uninformative for motility.

This dual-mechanism model is most vivid in children. The overall cohort had a median age of 31 years (IQR 22–50), but 130 patients (12.0%) were younger than 18 years, and the youngest was 11 months old. Pediatric orbital floor fractures characteristically present as ‘white-eyed’ trapdoor fractures: a linear, minimally displaced fracture with little or no periorbital ecchymosis and an often unremarkable-appearing CT, in which the inferior rectus muscle or its fascial sleeve is nonetheless incarcerated in the fracture line, producing severe motility restriction, marked diplopia, and occasionally an oculocardiac reflex (bradycardia, nausea, and vomiting) that constitutes a surgical emergency regardless of how small the radiographic defect appears. Contemporary pediatric series bear this out: in a multi-center cohort of trapdoor fractures, outcome turned on what had become entrapped and on how quickly it was released, with surgery within 48 h associated with markedly better recovery of ocular motility [17], and a more recent cohort with systematic review found the same advantage for repair within 24 h [18]. This phenotype is the clinical archetype of the structural-functional dissociation reported here: the smallest, most benign-appearing defects can carry the greatest functional risk. It is consistent with the companion analysis of this registry addressing pediatric inferior rectus entrapment, noted below, in which entrapment was concentrated at ages 8–11 years and was not associated with CT defect area, mirroring the null functional signal reported in the present all-ages analysis.

This study has several limitations. It is retrospective and single-center. Ocular assessment did follow an institutional protocol requiring joint evaluation by maxillofacial surgery and ophthalmology (oculoplastics) before, and for operated patients after, intervention, with diplopia registered only on concordance between the two specialties; however, the registry does not capture a standardized gaze-specific grading scale, forced-duction testing, or the exact examination interval in hours or days, and the timing of enophthalmos assessment relative to injury was likewise not fixed beyond this protocol structure. A two-dimensional elliptical area is a crude summary of a three-dimensional defect and does not capture gap geometry, herniation height, or muscle position, which the literature suggests are the measurements that matter functionally; in complex multi-wall fractures involving both the orbital floor and medial wall, this planar approximation is expected to systematically underestimate the true surface area spanning two perpendicular anatomical planes—an underestimation demonstrated directly when orbital floor defects are measured in two rather than three dimensions [19]—and area estimates in this subgroup should be interpreted with this in mind. We similarly did not model defect location within the orbital floor (anterior versus posterior) or the specific contribution of medial wall extension beyond the categorical fracture-pattern adjustment; fractures of similar total area but different location or wall involvement may carry different functional risk [15], and this remains an important direction for more granular morphometric analysis of this registry. Hypoglobus was recorded only in operated patients, so its estimate applies to that subgroup. The registry spans more than two decades (2001–2023), during which CT acquisition technology changed substantially, from thick-slice (3–5 mm) helical scans with limited multiplanar reformation in the early 2000s to sub-millimeter isotropic acquisition with routine coronal and sagittal reconstruction today, a shift that measurably improves the precision of volumetric defect assessment [20]; we therefore compared the 2001–2010 and 2011–2023 subperiods directly (Table 3). Both the structural associations and the functional nulls were reproduced within each era, and no area-by-era interaction approached significance, which argues against scan-era measurement precision as an explanation for the dissociation reported here. Any residual imprecision in the earlier era would in any case be expected to bias associations toward, rather than away from, the null. Finally, the analysis is cross-sectional with respect to phenotype and cannot establish that a larger defect causes herniation or enophthalmos.

The registry that supports this analysis has also supported three companion manuscripts, addressing the epidemiology of the full series, the age distribution of inferior rectus entrapment in children, and postoperative diplopia trajectories in operated patients. Those analyses use different units, denominators, and endpoints, and no table, figure, or result is shared with the present study; the overlap is declared to the editor.

Prospective work should pair standardized gaze-specific motility assessment with three-dimensional morphometry, including gap width, herniation height, and inferior rectus position, and should record the timing of globe-position assessment. Automated segmentation of orbital fractures is now feasible and would make such three-dimensional morphometry practical at scale [21]. The value of the defect area likely lies in combination with those measurements rather than as a stand-alone index of severity.

## 5. Conclusions

In a cohort of 1084 patients, orbital defect area was strongly associated with content herniation and globe-position abnormality but showed little to no association with extraocular movement abnormality or preoperative diplopia. In this cohort, defect magnitude reflected structural displacement rather than ocularmotor dysfunction; orbital fracture severity should not be inferred from defect size alone, and ocular motility must be assessed clinically regardless of defect size.

## Figures and Tables

**Table 1:** Cohort characteristics and phenotype prevalence by quartile of estimated orbital defect area (*n* = 1083 with measurable area).

Characteristic	Q1 (1.0–82.6 mm^2^)	Q2 (82.9–143.9 mm^2^)	Q3 (144.3–227.7 mm^2^)	Q4 (228.2–438.3 mm^2^)
*n*	273	269	270	271
Content herniation, *n* (%)	95 (34.8)	143 (53.2)	181 (67.0)	195 (72.0)
Documented enophthalmos, *n* (%)	11 (4.0)	33 (12.3)	62 (23.0)	83 (30.6)
Hypoglobus, *n* (%) ^a^	2 (1.1)	4 (2.2)	13 (6.5)	17 (8.6)
EOM abnormality, *n* (%)	48 (17.6)	45 (16.7)	47 (17.4)	46 (17.0)
Preoperative diplopia, *n* (%)	45 (16.5)	47 (17.5)	45 (16.7)	49 (18.1)

^a^ Hypoglobus denominators are 177, 180, 201 and 198 because the field was recorded only in operated patients. EOM, extraocular movement.

**Table 2:** Adjusted associations between estimated orbital defect area and each phenotype.

Phenotype	*n* (Events)	aOR per + 50 mm^2^ (95% CI)	*p*	FDR *p*	Standardized Probability, 50 vs. 300 mm^2^	Risk Difference, Percentage Points (95% CI)	AUC (95% CI)
Content herniation	1083 (614)	1.37 (1.28–1.48)	<0.001	<0.001	40.6% vs. 76.6%	+35.9 (28.5 to 43.4)	0.670 (0.636–0.703)
Documented enophthalmos	1082 (189)	1.45 (1.33–1.59)	<0.001	<0.001	7.4% vs. 33.7%	+26.3 (20.0 to 32.6)	0.704 (0.662–0.740)
Hypoglobus ^a^	756 (36)	1.48 (1.24–1.78)	<0.001	-	1.6% vs. 9.9%	+8.3 (4.2 to 12.5)	0.719 (0.635–0.799)
EOM abnormality	1083 (186)	1.00 (0.92–1.09)	0.944	0.944	17.1% vs. 17.3%	+0.2 (−5.8 to 6.2)	0.500 (0.455–0.544)
Preoperative diplopia	1083 (186)	1.01 (0.92–1.10)	0.849	0.944	16.9% vs. 17.5%	+0.6 (−5.5 to 6.6)	0.508 (0.460–0.553)

Logistic regression, one observation per patient, adjusted for age, sex, fracture pattern, associated facial fracture and mechanism of injury. ^a^ Secondary phenotype, restricted to patients in whom the field was recorded; not included in the false discovery rate family. aOR, adjusted odds ratio; AUC, area under the receiver operating characteristic curve; CI, confidence interval; EOM, extraocular movement; FDR, false discovery rate.

**Table 3:** Sensitivity analyses, adjusted odds ratio per +50 mm^2^ (95% CI).

Phenotype	Primary (*n* = 1083)	Isolated Floor (*n* = 655)	Single-Record Patients	All 1103 Injuries, Clustered	2001–2010 (*n* = 392) a	2011–2023 (*n* = 691) a	Threshold ≥ 200 mm^2^
Content herniation	1.37 (1.28–1.48)	1.42 (1.29–1.57)	1.56 (1.41–1.72)	1.37 (1.26–1.48)	1.32 (1.16–1.49)	1.44 (1.31–1.59)	2.51 (1.90–3.31)
Documented enophthalmos	1.45 (1.33–1.59)	1.40 (1.25–1.57)	1.31 (1.16–1.47)	1.46 (1.34–1.59)	1.47 (1.27–1.71)	1.47 (1.31–1.65)	2.53 (1.83–3.51)
Hypoglobus	1.48 (1.24–1.78)	1.45 (1.15–1.83)	1.45 (1.08–1.94)	1.46 (1.24–1.73)	1.37 (1.05–1.80)	1.60 (1.21–2.11)	4.12 (1.99–8.54)
EOM abnormality	1.00 (0.92–1.09)	1.01 (0.90–1.13)	1.00 (0.89–1.13)	1.00 (0.92–1.09)	0.94 (0.80–1.12)	1.05 (0.95–1.17)	1.10 (0.78–1.56)
Preoperative diplopia	1.01 (0.92–1.10)	1.04 (0.93–1.16)	1.02 (0.91–1.14)	1.01 (0.93–1.10)	1.05 (0.89–1.22)	1.01 (0.91–1.13)	1.16 (0.83–1.63)

The threshold column reports the adjusted odds ratio for a defect of 200 mm^2^ or larger versus smaller, not a per 50 mm^2^ estimate. a Scan era columns are a post hoc sensitivity analysis added at revision; no area-by-era interaction reached significance (all *p* ≥ 0.35). CI, confidence interval; EOM, extraocular movement.

## Data Availability

The anonymized data supporting the reported results are available from the corresponding author on reasonable request, subject to institutional data-sharing regulations and ethical approval.

## Supplementary Materials

The following supporting information can be downloaded at: https://www.mdpi.com/article/10.3390/diagnostics16183043/s1. Table S1: Adjudication of repeated registry records; Table S2: Registry coding conventions requiring explicit handling; Table S3: Restricted cubic spline tests for each phenotype.

## References

[1] Pandya R.P., Deng W., Hodgson N.M. (2023). Current guidelines and opinions in the management of orbital floor fractures. Otolaryngol. Clin. N. Am..

[2] Patel S., Shokri T., Ziai K., Lighthall J.G. (2022). Controversies and contemporary management of orbital floor fractures. Craniomaxillofac. Trauma Reconstr..

[3] Wevers M., Strabbing E.M., Engin O., Gardeniers M., Koudstaal M.J. (2022). CT parameters in pure orbital wall fractures and their relevance in the choice of treatment and patient outcome: A systematic review. Int. J. Oral Maxillofac. Surg..

[4] Hashem A.M., MacKenzie J., Flores A., Nikolli K., Rayham J., Chamma B., Papay F. (2026). Radiographic indications for orbital blowout fracture reconstruction: A critical review of 2 decades of literature. Plast. Reconstr. Surg. Glob. Open.

[5] Lentskevich M.A., Nguyen A., Choudhary A., Obaid O., Purnell C.A. (2025). What computed tomography findings are predictive of posttraumatic enophthalmos in orbital fractures?. Plast. Reconstr. Surg..

[6] Ploder O., Klug C., Voracek M., Burggasser G., Czerny C. (2002). Evaluation of computer-based area and volume measurement from coronal computed tomography scans in isolated blowout fractures of the orbital floor. J. Oral Maxillofac. Surg..

[7] Kunz C., Sigron G.R., Jaquiery C. (2013). Functional outcome after non-surgical management of orbital fractures—The bias of decision-making according to size of defect: Critical review of 48 patients. Br. J. Oral Maxillofac. Surg..

[8] Shah H.A., Shipchandler T.Z., Sufyan A.S., Nunery W.R., Lee H.B.H. (2013). Use of fracture size and soft tissue herniation on computed tomography to predict diplopia in isolated orbital floor fractures. Am. J. Otolaryngol..

[9] Bruneau S., De Haller R., Courvoisier D.S., Scolozzi P. (2016). Can a specific computed tomography-based assessment predict the ophthalmological outcome in pure orbital floor blowout fractures?. J. Craniofac. Surg..

[10] Basta M.N., Rao V., Roussel L.O., Crozier J.W., Liu P.Y., Woo A.S. (2021). Refining indications for orbital floor fracture reconstruction: A risk-stratification tool predicting symptom development and need for surgery. Plast. Reconstr. Surg..

[11] Furuta M., Yago K., Iida T. (2006). Correlation between ocular motility and evaluation of computed tomography in orbital blowout fracture. Am. J. Ophthalmol..

[12] Oku H., Watanabe A., Rajak S.N., Nakayama T., Yoneda A., Yoshii K., Sotozono C. (2025). Factors associated with diplopia before and after orbital blowout fracture reconstruction. Br. J. Ophthalmol..

[13] Schonegg D., Wagner M., Schumann P., Essig H., Seifert B., Rucker M., Gander T. (2018). Correlation between increased orbital volume and enophthalmos and diplopia in patients with fractures of the orbital floor or the medial orbital wall. J. Craniomaxillofac. Surg..

[14] von Elm E., Altman D.G., Egger M., Pocock S.J., Gotzsche P.C., Vandenbroucke J.P. (2007). The Strengthening the Reporting of Observational Studies in Epidemiology (STROBE) statement: Guidelines for reporting observational studies. Lancet.

[15] Chen H., Bao Y., Chen Y., Li Y., Wu P., Fan G., Zhang J. (2025). Predictors of diplopia following orbital fractures based on anatomical location, a retrospective cohort study. Head Face Med..

[16] Isa M.F., Nazimi A.J., Ramli R. (2025). Predictor of diplopia in pure orbital blowout fracture: A 14-year retrospective analysis. J. Stomatol. Oral Maxillofac. Surg..

[17] Eshraghi B., Khademi B., Rafizadeh S.M., Noorshargh P., Attar A., Shahsavari A., Ghorbani S. (2025). Outcomes and prognostic factors in pediatric orbital trapdoor fracture: A multi-center study. Oral Maxillofac. Surg..

[18] Takamura N., Sato A., Matsunaga H., Imai T., Yusa Y., Hara K., Hayashi M., Ishi S., Kurosawa K., Imai Y. (2026). Surgical repair within 24 hours improves ocular motility in pediatric orbital fractures with muscle entrapment: Cohort and systematic review. Plast. Reconstr. Surg..

[19] Taxis J., Ungerboeck L., Gehrking M.R., Motel C., Wurm M., Eckert A.W., Spanier G., Nieberle F., Platz Batista da Silva N., Ludwig N. (2023). Two-dimensional post-traumatic measurements of orbital floor blowout fractures underestimate defect sizes compared to three-dimensional approaches. Tomography.

[20] Kormi E., Lusila N., Mäntynen P., Männistö V., Suojanen J. (2026). Volumetric assessment of blow-out fractures with automated segmentation benefits thinner computed tomography slice thickness: A retrospective case-control study. J. Craniofac. Surg..

[21] Bao X.-L., Zhan X., Wang L., Zhu Q., Fan B., Li G.-Y. (2023). Automatic identification and segmentation of orbital blowout fractures based on artificial intelligence. Transl. Vis. Sci. Technol..

