# Supplementary material for: Orbital Defect Size Reflects Structural Displacement but Not Ocular Motor Dysfunction: A Cohort Study of 1084 Patients
Source: Diagnostics (Basel). 2026 Sep 19;16(18):3043. doi: 10.3390/diagnostics16183043 (PMC13605676; doi:10.3390/diagnostics16183043)
Supplement: Supplementary file 1 [file diagnostics-16-03043-s001.zip › diagnostics-4571844-supplementary.pdf]

Supplementary Materials

Orbital Defect Size Reflects Structural Displacement but Not Ocular Motor Dysfunction: A Cohort Study of 1084 Patients

Table S1. Adjudication of repeated registry records.

Step	Rows	Patients	Injuries
Source registry file	1489	1084	-
After removal of 26 exact duplicate rows	1463	1084	-
After adjudication by patient, date and laterality	1463	1084	1103
Analytic cohort: index injury per patient	-	1084	1084
With measurable defect dimensions	-	1083	1083

Rows sharing a patient identifier were assigned to the same injury when they carried the same recorded age, or dates within 90 days of one another; rows recording opposite orbits on the same date were treated as one bilateral injury. Records with 0 × 0 mm entries were treated as non-measurable.

Table S2. Registry coding conventions requiring explicit handling.

Field and value	Records	Handling
Enophthalmos field, uninterpretable mark	7	Treated as missing, not assigned to either category
Exophthalmos documented	2	Not counted as enophthalmos
Hyperglobus documented	10	Not counted as hypoglobus
Hypoglobus marked not applicable	402	Treated as missing; all in non-operated patients

Table S3. Restricted cubic spline tests for each phenotype.

Phenotype	Overall association P	Non-linearity P
Content herniation	< 0.001	< 0.001
Documented enophthalmos	< 0.001	0.032
Hypoglobus	< 0.001	0.594
EOM abnormality	0.355	0.197

Phenotype	Overall association P	Non-linearity P
Preoperative diplopia	0.060	0.025

Likelihood ratio tests against a model without defect area (overall) and against a model with linear defect area (non-linearity), each adjusted for age, sex, fracture pattern, associated facial fracture and mechanism of injury. The non-linearity term for preoperative diplopia reached nominal significance in the absence of any overall association and is most plausibly noise. EOM, extraocular movement.
